# Glycans affect DNA extraction and induce substantial differences in gut metagenomic studies

**DOI:** 10.1038/srep26276

**Published:** 2016-05-18

**Authors:** Emmanouil Angelakis, Dipankar Bachar, Bernard Henrissat, Fabrice Armougom, Gilles Audoly, Jean-Christophe Lagier, Catherine Robert, Didier Raoult

**Affiliations:** 1 URMITE, UM63, CNRS 7278, IRD 198, Inserm 1095, Aix Marseille Université,13005 Marseille, France; 2 Centre National de la Recherche Scientifique, UMR 7257, Aix-Marseille Université, Marseille, France

## Abstract

Exopolysaccharides produced by bacterial species and present in feces are extremely inhibitory to DNA restriction and can cause discrepancies in metagenomic studies. We determined the effects of different DNA extraction methods on the apparent composition of the gut microbiota using Illumina MiSeq deep sequencing technology. DNA was extracted from the stool from an obese female using 10 different methods and the choice of DNA extraction method affected the proportional abundance at the phylum level, species richness (Chao index, 227 to 2,714) and diversity (non parametric Shannon, 1.37 to 4.4). Moreover DNA was extracted from stools obtained from 83 different individuals by the fastest extraction assay and by an extraction assay that degradated exopolysaccharides. The fastest extraction method was able to detect 68% to 100% genera and 42% to 95% species whereas the glycan degradation extraction method was able to detect 56% to 93% genera and 25% to 87% species. To allow a good liberation of DNA from exopolysaccharides commonly presented in stools, we recommend the mechanical lysis of stools plus glycan degradation, used here for the first time. Caution must be taken in the interpretation of current metagenomic studies, as the efficiency of DNA extraction varies widely among stool samples.

The gastrointestinal tract harbors >10^14^ microorganisms, and different species and quantities of bacteria are present at different locations along the digestive tract due to major variations in environmental niches^1^,^2^. Studies involving the amplification and sequencing of 16S rRNA as well as metagenomic analyses have dramatically expanded our knowledge of the diversity of the human gut microbiome. Several factors including diet, host genetic and familial relationships, varying cultural traditions and geography, age, obesity, metabolic syndrome, and type II diabetes, cardiovascular disease, disturbances produced by antibiotics, inflammatory bowel disease, irritable bowel syndrome and necrotizing enterocolitis have been associated with gut microbiota modifications^3^,^4^. Most methods of deriving the taxonomic community composition have been based on PCR-denaturing gradient gel electrophoresis, 16S rRNA gene sequencing and the HITChip microarray^2^,^4^. Moreover, culture-dependent methods for exploring gut microbiota are critical, as the advent of molecular tools has revolutionized our ability to investigate this ecosystem^5^.

Technical aspects have been shown to be important for the comparison and the analysis of the gut microbiota^6^. As a result, limited comparability in human microbiome data sets often results from differences in sample preservation, DNA isolation protocols as well as from sequencing of different 16S rRNA gene regions. The DNA extraction method used has an impact on microbial community representation^7^,^8^,^9^,^10^,^11^,^12^. However, the relative efficacy of these DNA extraction methods and the optimum weight range of samples for extraction require further evaluation. A meta-analysis assessing the effect size of technical differences on data comparability showed that samples rather cluster by study or the methods applied than by the parameter of interest^4^. Indeed DNA extraction methods cause bias in PCR amplification caused by inhibitors present in fecal specimens, such as bile salts and complex polysaccharides, or because of the amount of fecal specimen used in the extraction process^13^,^14^. In addition, the disruption and/or lysis of the bacterial membranes can be expected to cause bias for specific bacterial taxa due to differences in cell wall structure and integrity. Various methods have been developed to remove or inactivate inhibitors in stool, and it is critical to optimize DNA extraction methods to obtain accurate results on the composition of gut microbiota^15^. For example, DNA from Gram-positive bacteria present in feces is more efficiently extracted if a sample has been frozen^16^. Although PCR inhibitors are common in stool samples, little attention has been paid to the potential biases introduced by exopolysaccharides produced by the microorganisms of the gut microbiota^13^. Indeed *Escherichia coli*^17^ and Enterococci^18^ that are commonly presented in the gut microbiota have been associated with the production of exopolysaccharides. As a reference centre for Whipple’s Disease we commonly test stool samples for *Tropheryma whipplei*. These bacteria produce extracellularly glycoproteins that affect the sensitivity of molecular assays^19^. Indeed, exopolysaccharides produced by bacterial species and present in feces are extremely inhibitory to DNA restriction and modifying enzymes^20^,^21^. To allow a good liberation of DNA from stool exopolysaccharides, we developed a variation of the Qiagen stool procedure [QIAamp® DNA Stool Mini Kit (Qiagen, Courtaboeuf, France) based on glycan degradation. We then used the Illumina MiSeq deep sequencing platform to determine the effects of this extraction method on gut microbiota community composition comparing to other DNA extraction methods and variations of the Qiagen stool kit.

## Results

### 16S rRNA gene sequencing of the faecal microbial community of an obese subject using 10 different DNA extraction protocols

**Figure 1:**
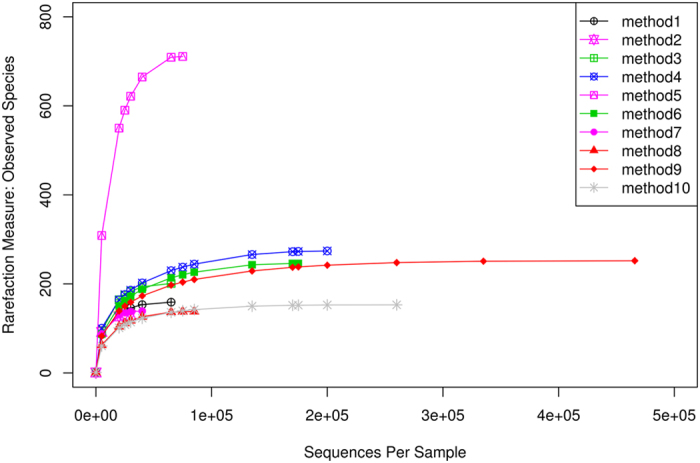
Rarefaction curves relating the sequencing effort to the number of putative species in the samples.

We analyzed a total of 1,443,537 high-quality sequences of 411 bp in length. The number of OTUs for all reads was between 167 to 2,127 for each extraction method (Supplementary Table 1). The number of OTUs obtained using the different stool extraction methods was found by rarefaction curves, which relate the sequencing effort to the number of putative species in samples (Fig. 1). The lowest number of reads 28,277 produced by method 2 and the highest number of reads 465,988 produced by method 9 (Supplementary Fig. 1). The analysis of the sequences was stopped for each extraction method when the number of OTUs reached a plateau. We obtained the highest number of OTUs per number of reads with extraction method 5 (Supplementary Fig. 2). In contrast, we obtained a high number of OTUs with extraction methods 9 and 10 but analyzed considerably more sequences. Lower numbers of OTUs were obtained with the other extraction methods, with extraction method 8 resulting in the lowest species diversity.

### Comparison of DNA extraction methods

**Figure 2:**
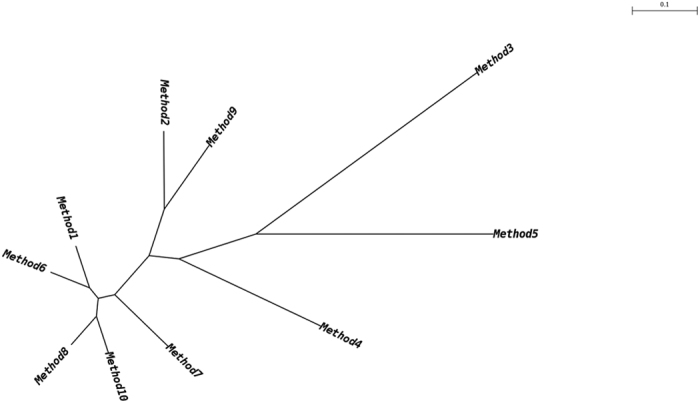
Consensus dendrogram illustrating the similarity of the microbial communities obtained using different DNA extraction methods.

We used a principal coordinate analysis and principal component analysis to compare and contrast the apparent composition of microbial communities obtained with the different DNA extraction methods (Supplementary Figs 3 and 4). We found that data points did not always group closely together, indicating that the methods did not retrieve the same components of the community with equal efficiency. As a result, the apparent bacterial community composition varied among the extraction methods. Close grouping of data points was observed for most of the extraction methods tested. In contrast, data points for extraction methods 5 and 3 were much more widely spread than for the other methods. Spearman’s rank and Pearson’s correlations between the different DNA extraction methods revealed good correlations between the apparent microbial composition data from the 8 other methods (Supplementary Table 2). As a result, Pearson’s correlation coefficient (*r*) ranged from 0.99 to 0.05, the latter of which was the lowest Pearson’s correlation observed and was between extraction methods 2 and 3. In contrast, methods 8 and 10 correlated well, with the correlation score being at 0.99. Our results were also confirmed by a consensus dendrogram illustrating the similarity of the microbial communities obtained by the different DNA extraction methods (Fig. 2), with apparent similarity using extraction methods 1, 6, 7, 8 and 10.

### Richness by different extraction methods

We compared OTUs at the phylum level to determine factors contributing to the quantitative differences between the eight extraction methods. Most sequences belonged to *Firmicutes*, *Actinobacteria* and *Verrucomicrobia*, with the remainder being distributed among *Proteobacteria*, *Bacteroidetes*, *Chloroflexi*, *Deinococcus*-*Thermus*, *Euryarchaeota* and *Fusobacteria*. The choice of DNA extraction method affected the abundance of bacterial groups at various taxonomic ranks, and each extraction method produced different proportions at the phylum level (Supplementary Fig. 5). At this level, Firmicutes was more abundant in the results from extraction methods 3 and 4 relative to the other methods. *Verrucomicrobia* was relatively more abundant in methods 1, 6, 8 and 9, whereas *Actinobacteria* was relatively more abundant in methods 2, 5 and 10. DNA extraction method 5 was the most effective for the detection of *Proteobacteria* OTUs, whereas DNA extraction methods 3, 4, 5 and 10 were the most effective for the detection of *Bacteroidetes* OTUs. As a result, we found that the ratio of *Firmicutes*/*Bacteroidetes* varied dramatically among the different extraction methods, with the highest ratio obtained from method 1 and the lowest from method 5 (Table 1). Finally, large variations were also found in the sequences obtained for *E. coli*, *Bifidobacterium* spp., *Lactobacillus* spp., *Prevotella* spp., *Staphylococcus* spp. and *Enterococcus* spp. (Table 1).

**Figure 3:**
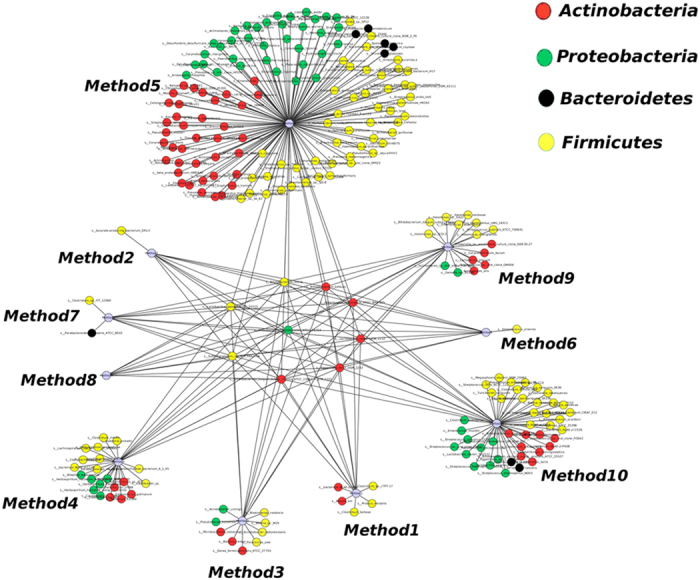
Network of species only detected by each extraction method.

Difference were obtained for the species richness and diversity, as estimated by the Chao1 index, and biodiversity, as assessed using a nonparametric Shannon index, for the different DNA extraction methods while taking into account an OTU distance unit cutoff of 3 as previously described^22^ (Supplementary Table 1). Only 9 species were detected by all the extraction methods used in this study (Fig. 3). The DNA extraction method 5 had the highest microbial richness and diversity and 129 species were only detected by this method (Fig. 3). In contrast the extraction method 8 detected no species that was not found by the use the other DNA extraction methods (Fig. 3).

Finally, when we compared the number of genera and species detected by each extraction method, we found that DNA extraction method 5 detected the 77% of genera and the 70% of species identified by all DNA extraction assays (Supplementary Table 3).

### Application of our assays to other stool samples

**Figure 4:**
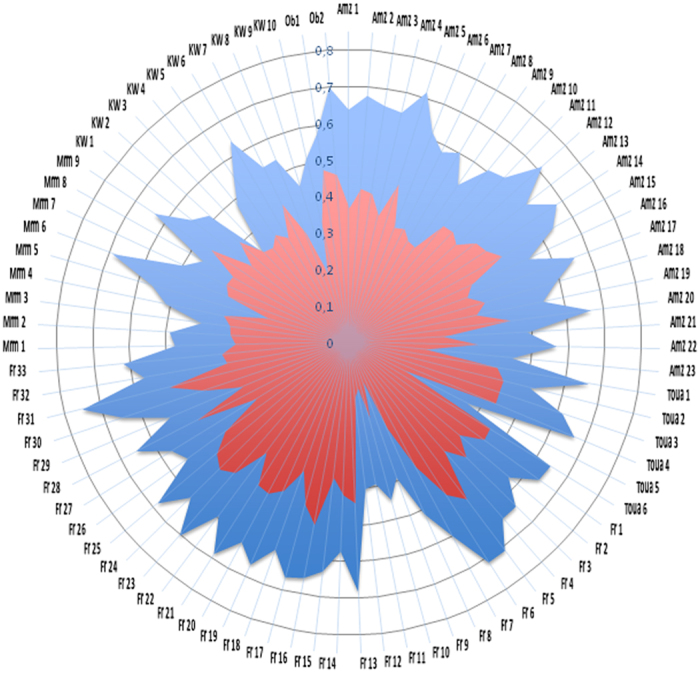
Correlation coefficient of species detected by DNA extraction methods 1 and 5 for 83 stool samples. Amz, individuals from Amazone; Toua, Touareg individuals; Fr, French individuals; Mrm, Marasmus individuals; KW, kwashiorkor individuals; Ob, obese individuals Blue color, genera coefficient; red color, species coefficient. Correlation coefficient was estimated for each sample using the number of shared species between methods over the total number of species/genus identified.

We tested stools from individuals with different origins including 33 stools from normal-weight French individuals, 23 stools from normal-weight volunteers from Amazon, 6 Touaregs, 9 from marasmus individuals, 10 from individuals with kwashiorkor and 2 from obese individuals. All samples were tested using the fastest DNA extraction assay (method 1) and by the most effective assay that used mechanical lysis of stools plus glycan degradation (methods 5). We analyzed a total of 16,203,449 high-quality sequences of 411 bp in length. The number of OTUs for all reads was between 140 to 16,012 for each extraction method (Supplementary Table 4). Coefficient of correlation between the 2 DNA extraction methods revealed large differences with regard to the number of genera and species detected for each stool sample (Fig. 4). Indeed, with DNA extraction method 1, we detected more phyla than with extraction method 5 for 30 stools (36%) and less phyla for 39 stools (47%); for 16 stools (19%), both assays resulted in an equal number of plyla (Supplementary Fig. 6). Moreover, with DNA extraction method 1, we detected more genera than with extraction method 5 for 28 stools (35%) and less genera for 33 stools (41%); for 20 stools (25%), both assays resulted in an equal number of genera (Supplementary Table 4 and Supplementary Fig. 7). Differences were also found in the percentage of amount between both methods (Supplementary Table 5) and when we compare the 3 obese individuals (Supplementary Fig. 8). In addition, with DNA extraction method 1, we detected more species than with method 5 for 39 stools (48%) and less species for 37 stools (46%); for 5 stools (6%), both assays resulted in an equal number of species (Supplementary Table 4 and Supplementary Fig. 9). Moreover, the microbial richness estimated by the Chao1 index and biodiversity, as assessed using a nonparametric Shannon index, revealed large differences between the two DNA extraction methods for the stools samples tested (Supplementary Table 4). We then determined the number of phyla, genera and species detected by the two techniques (Supplementary Table 4). Extraction method 1 was able to detect 68% to 100% genera and 42% to 95% species whereas extraction method 5 was able to detect 56% to 93% genera and 25% to 87% species.

## Discussion

The present study shows that different DNA extraction assays result in variable Illumina deep sequencing results. We obtained similar results by our analyses before and after normalization. None of the different DNA extraction methods that were used resulted in 100% comparable bacterial community compositions. Indeed, for the human fecal sample from an obese individual, only a few DNA extraction methods were similar enough to allow for the direct comparison of most bacterial community parameters. Extraction method 5 resulted in a higher microbial richness and diversity for the sample from the obese individual compared to the other methods. However, it is apparent that even this extraction method was able to detect only 73% of all identified OTUs. Our current strategy for the exploration of the gut microbiota is based on the combination of different DNA isolation methods, and we use extraction method 5, which had the best results for the obese stool sample, and extraction method 1, which was the easier and faster method. Based on the results from the 83 stools we tested, we believe that differences in stool composition and molecular assays inhibitors in each stool were responsible for the high variations in the results obtained by the various extraction methods used. The two DNA isolation methods (1 and 5) presented different sensitivities at the genus and species levels, even for the stool samples from individuals of the same origin. As a result, the combination of different extraction methods is critical for future studies on the composition and diversity of the gut microbiota and can result in a notable increase in the microbial richness and diversity of stools.

Feces constitute complex biological samples, causing problems in molecular assays due to the presence of numerous types of bacteria and different types of food degradation products^23^. Previously, Peng *et al.*^24^ used direct boiling of the stools to improve the efficiency of DNA extraction and they found similar community structures by sequencing after extraction using direct boiling or after extraction with most of the commercial kit methods. However we cannot compare our results to that of Peng *et al.*^24^ because we amplified a different 16S region and we used a different cutoff for clustering. In the present study, the combination of the mechanical lysis of stools by FastPrep® with enzymes that break down glycans resulted the best results for extraction method 5. This increase in microbial richness and diversity was not found when we used either the mechanical lysis of stools by FastPrep® or enzymes degrading bacterial glycans alone. We believe that the addition of cellulase facilitated the breakdown of bacterial exopolysaccharides. We believe that the association of these two processes provided the best action for freeing DNA from the sample. Complex glycans in fecal samples originating from vegetable consumption in the diet have been proposed as PCR inhibitors^23^,^25^, and it was proposed that polysaccharides may disturb the enzymatic process by mimicking the structure of nucleic acids^13^. Previous studies showed that bacteria could remain undetected in stools, even at very high concentrations, because of these PCR inhibitors^7^,^23^,^26^. As a result, the need for internal controls to detect PCR inhibitors when gut microbiota samples are analyzed can be critical.

Since 2000, large-scale 16S rRNA and metagenomic studies have been commonly used to explore gut microbiota. Many of these studies have targeted the V6 region of the 16S ribosomal subunit, which is known to be the most variable region and is commonly utilized for exploring gut microbiota diversity^27^. We found that gut microbiota composition and relative phylum abundance were both dramatically biased by the DNA extraction method used (Table 1). The fact that we detected significant more proteobacteria by the DNA extraction method 5 in the obese sample can possibly explained by the exopolysaccharide production of these microorganisms in the gut microbiota^17^,^28^. Our results corroborate earlier reports that DNA isolation methods introduce bias into studies of gut microbiota^8^,^16^,^29^,^30^. However, it is possible that results can be also influenced by the different origin of the examined population^31^,^32^,^33^ and by inter-individual variations on the composition of the gut microbiota^34^.

In conclusion, DNA extraction protocols critically affect the results of studies on gut microbiota. Inter individual variations in association with inhibitors of molecular assays that are very common in stool samples, critically affect the results on the composition of the gut microbiota. The comparison of different studies exploring the gut microbiota can lead to incorrect conclusions regarding probably the association of the gut microbiota with other syndromes as well due to the use of different DNA extraction methods. Extracellularly complex polysaccharides in fecal samples can bias the results on the composition of the gut microbiota and we believe the mechanical lysis of stools plus glycan degradation can break down glycans allowing a very good liberation of DNA.

## Materials

### Samples

We tested stools from 3 obese individuals and from individuals with different origin including volunteers from Amazon, Touaregs, French, marasmus and kwashiorkor. The exclusion criteria were, a history of colon cancer, bowel inflammatory disease, acute or chronic diarrhea in the previous 4 weeks, and antibiotic treatment in the 6 months before fecal sampling. The clinical data (gender, date of birth, clinical history, weight, height and antibiotic use) were recorded using a standardized questionnaire. All patients gave their informed and signed consent. Both this study and the assent procedure were approved by the Ethics Committee of the Institut Fédératif de Recherche IFR48, Faculty of Medicine, Marseille, France, and the agreement of the ethics committee of the IFR48 (Marseille, France) was obtained under reference 09, 10, 11, 12, 13, 14, 15, 16, 17, 18, 19, 20, 21, 22. All the methods of this study were carried out in accordance with the approved guidelines.

### DNA extraction methods

We selected 10 different DNA extractions methods that were commercially available, published in the literature or used in our laboratory. DNA was extracted from the same stool sample of a 36-year-old obese female with a body mass index (BMI) of 30.4 in duplicate for all methods. Extraction method 10 was tested only once because we did not have enough sample for a duplicate assay. In addition, we tested stool samples from 81 individuals by both extraction methods 1 and 5. The quantity, purity, integrity and size of DNA and its amenability to PCR amplification were assessed. The concentration of each DNA extraction was measured by a Qubit assay with the high sensitivity kit (Life technologies, Carlsbad, CA, USA) according to the Nextera XT DNA sample prep kit (Illumina) and dilution was performed to require 1ng of each metagenome as input for the paired end sequencing. DNA extracts were dispensed into 10- to 20-μl single-use aliquots and frozen at −20 °C to avoid repeat freeze-thaw cycles prior to downstream analyses.

### Extraction method 1

Fecal DNA was extracted from 0.25 g of stool using a Qiagen stool procedure [QIAamp® DNA Stool Mini Kit (Qiagen, Courtaboeuf, France)] according to the protocol described by Zoetendal *et al.*^35^.

### Extraction method 2

A total of 0.25 g of stool was lyophilized using a Lyovac GT2 (GEA Process Engineering, Maryland, USA). The lyophilized products were diluted in 500 μL of PBS and sonicated for 1 hour. Subsequently, 25 μl of trypsin (5%) was added to the sonicated products for 15 minutes at 37 °C. DNA was then extracted from the samples using extraction method 1^35^.

### Extraction method 3

A total of 0.25 g of stool was lyophilized using a Lyovac GT2. The lyophilized products were diluted in 12 mL of PBS, harvested and broken using a French press for 20 minutes. DNA was then extracted from the samples using extraction method 1^35^.

### Extraction method 4

Fecal DNA was extracted from 0.25 g of stool using the PowerBiofilm^TM^ DNA Isolation Kit (MOBIO laboratories, Carlsbad, USA), as described by the manufacturer^36^.

### Extraction method 5

We added 500 μL of PBS to 0.25 g of stool, which was then homogenized using FastPrep® (Biomedicals, Santa Ana, California, USA), as described by the manufacturer. Then, 200 μL of this mixture was centrifuged at 17000 rpm for 10 minutes. We removed the supernatant and resuspended the pellet in 20 μl of 10X Glycoprotein Denaturing Buffer (New England Biolabs). We denatured the glycoproteins by heating reaction at 100 °C for 10 minutes. We then added 160 μl of H_2_O and 40 μl of 10X G5 reaction buffer, followed by 5 μl of EndoHf (New England Biolabs, USA), 5 μl of cellulase (SIGMA, France) and 5 μl of PNGase F (SIGMA). The mixture was incubated overnight at 37 °C, after which the DNA was extracted using extraction method 1^35^.

### Extraction method 6

We added 500 μL of PBS to 0.25 g of stool and sonicated the sample for 2 hours. We then added 1 mmol of EDTA and 0.1 mmol of sodium dodecyl sulfate, and the DNA was extracted from this mixture using extraction method 1^35^.

### Extraction method 7

A total of 0.25 g of stool was lyophilized using a Lyovac GT2. The lyophilized products were diluted in 1 mL of PBS containing 10 μl of Tween (1%). This mixture was placed in liquid nitrogen for 30 minutes and then crushed. We added 500 μl of PBS and sonicated for 1 hour. Subsequently, we added 25 μl of trypsin (5%), and the mixture was incubated for 15 minutes at 37 °C. DNA was extracted from this mixture using extraction method 1^35^.

### Extraction method 8

To 0.25 g of stool, we added 500 μL of PBS, 1 mmol of EDTA and 0.1 mmol of sodium dodecyl sulfate as well as the enzymes EndoHf (5 μl), cellulase (5 μl), PNGase (5 μl) and proteinase K (12 μl). This mixture was incubated for 2 hours at 37 °C, and the DNA was then extracted using extraction method 1^35^.

### Extraction method 9

We added 500 μL of PBS to 0.25 g of stool, which was then homogenized using FastPrep® (Biomedicals, Santa Ana, California, USA), as described by the manufacturer. The DNA was extracted using extraction method 1^35^.

### Extraction method 10

An aliquot (~500 mg) of each sample was suspended, while frozen, in a solution containing 500 μl of extraction buffer [200 mM Tris (pH 8.0), 200 mM NaCl, 20 mM EDTA], 210 μl of 20% SDS, 500 μl of a mixture of phenol:chloroform:isoamyl alcohol (25:24:1, pH 7.9) and 500 μl of a slurry of 0.1-mm diameter zirconia/silica beads (BioSpec Products, Bartlesville, OK). The microbial cells were subsequently lysed by mechanical disruption with a FastPrep® on high for 2 min at room temperature, followed by extraction with phenol:chloroform:isoamyl alcohol and precipitation with isopropanol, as previously described^37^.

### Illumina MiSeq deep sequencing

PCR amplification from a genomic DNA template was performed using primers, with overhanging adaptors, based on the surrounding conserved regions V3-V4 (FwOvAd_341F TCGTCGGCAGCGTCAGATGTGTATAAGAGACAGCCTACGGGNGGCWGCAG; ReOvAd_785RGTCTCGTGGGCTCGGAGATGTGTATAAGAGACAGGACTACHVGGGTATCTAATCC). The samples were amplified individually for the 16S “V3-V4” regions using Taq Phusion and visualized on the Caliper LabchipII device with a DNA 1-K Labchip. After purification with AMPure beads, the DNA concentrations were measured using the high-sensitivity Qubit technology. Using a subsequent limited-cycle PCR with 1 ng of each PCR product, Illumina sequencing adapters and dual-index barcodes were added to each amplicon. After purification with AMPure beads, the libraries were then normalized according to the Nextera XT protocol.

The samples were pooled into a single library for MiSeq sequencing. The pooled library containing indexed amplicons was loaded onto the reagent cartridge and then onto the instrument along with the flow cell. Automated cluster generation and paired-end sequencing with dual index reads was performed in a single 39-hour run using 2 × 250-bp read lengths. The global cluster density and global passed filter per flow cell was calculated on the instrument. The MiSeq Reporter software calculated the percentage of indexing and cluster passed filter (PF) for each amplicon or library. The raw data were configured in fastq files for R1 and R2 reads.

### Treatment of raw sequences: assembly and filtering

Paired-end raw reads from the fastq files produced by Illumina MiSeq were first assembled using PANDAseq^38^, which has the ability to filter out low-quality reads during the assembly of paired-end reads. In the next filtering step, the tags were extracted from the fasta file produced by PANDAseq only if they contained the corresponding primers. Finally, the sequences produced by the previous steps were filtered again by deleting sequences containing “N” and by removing sequences shorter then 200 nt. The primers were also removed from the sequences during this step. The high-quality sequences resulting from this strict filtering and cleaning process were then ready for further analysis.

### Dereplication, clustering and operational taxonomic unit extraction

The high-quality sequences from the previous steps were strictly dereplicated and sorted in order of decreasing abundance^39^. The sorted sequences were then clustered at 97% similarity. Next, operational taxonomic units (OTUs) were extracted from each cluster. These OTUs were the unique sequences from each cluster that had the maximum number of occurrences during PCR amplification^39^. Abundance information for each sequence was identified during dereplication using UCLUST bioinformatic tool^40^.

### Building the reference database

In this step, we constructed database of 16S sequences using release 115 of the SILVA SSU and LSU databases (http://www.arb-silva.de/download/archive)^41^. From this, a database of predicted amplicon sequences was built. Only SILVA SSU reference sequences containing the two primers from the PCR amplification were considered, and 3 differences between each primer and the SILVA SSU reference sequences were allowed^41^. Only sequences between the two primers from the SILVA reference database were extracted while building our local reference database. Ultimately, our reference database contained a total of 479,927 well-annotated sequences.

### Taxonomic assignments

Representative sequences of each OTU extracted in the previous steps were searched against our reference database using the Needleman–Wunsch global alignment algorithm^39^,^41^. The best matches, with greater than 80% similarity to each unique representative sequence, were then extracted from the reference database. These extracted reference sequences were sorted by decreasing percentage of similarity, and the fractional values were rounded to the nearest integer. The reference sequences with the highest percentage of similarity to the OTUs were used for taxonomic assignments, and the taxonomy of each rank was obtained by consensus when multiple results with the same percentage of similarity were present. For example, a tag with 98% similarity to the classes Gammaproteobacteria and Alphaproteobacteria was only assigned to the phylum Proteobacteria^39^. When similarity was 80% or less, the sequences were not assigned^39^. Finally, all tags were clustered into different taxon ranks according to the consensus taxonomy of the unique tags (representative of each OTU).

### Statistical analysis

Richness and biodiversity indices for the OTUs were calculated using the Mothur software package^22^, with its implementation of Chao1 and the non-parametric Shannon^42^ formula. Richness was estimated using the Chao1 index, whereas diversity, which depends on how uniformly the sequences are spread across different OTUs, was estimated using the non-parametric Shannon formula. Rarefaction curves were calculated and plotted with a combination of the mothur^22^ and R statistical software packages. A principal coordinate analysis (PcoA) at the genus level according to Bray-Curtis dissimilarities was carried out using QIIME^43^. Pearson’s correlations between the different DNA extraction methods were calculated at the genus level using R. These correlation matrixes were then converted into distance matrices, where distance = (1 − correlation). This distance matrix was used to generate a tree using the UPGMA algorithm^41^. ANOVA tests were used to identify significant differences between the measured parameters after the different DNA extraction methods. To compare the extraction methods, correlations between the different methods were calculated for relative abundance at the phylum, family and genus levels using nonparametric statistical methods (Kruskal-Wallis test). A coefficient of correlation between the extraction methods 1 and 5 was estimated for each sample using the number of shared species/genus between methods over the total number of species/genus identified. For data comparison, we used EpiInfo software, version 6.0 (Centers for Disease Control and Prevention, Atlanta, GA, USA). A *p* value < 0.05 was considered significant.

## Additional Information

**How to cite this article**: Angelakis, E. *et al.* Glycans affect DNA extraction and induce substantial differences in gut metagenomic studies. *Sci. Rep.* **6**, 26276; doi: 10.1038/srep26276 (2016).

## Supplementary Material

Supplementary Information

## Figures and Tables

**Table 1:** Bacteria previously associated with weight modifications and their detection by different extraction assays.

DNA extraction method	*Firmicutes*	*Bacteroidetes*	*Firmicutes*/ *Bacteroidetes* Ratio	*Escherichia coli*	Bifidobacteria	*Lactobacillus* sp.	*Staphylococcus* sp.	*Prevotella sp.*	*Ruminococcus sp.*	*Bacteroides sp.*	*Clostridium sp.*	*Enterococcus* sp.	Unassigned OTU
Method 1	18,882	8	2.360	21,101	253,077	9,498	229	0	280	5	18,519	548	252
Method 2	39,197	60	653	2,398	683,071	25,554	0	0	254	12	9,917	3,874	108
Method 3	79,285	260	305	436	19,237	2,563	1,625	235	27	12	184	766,116	110
Method 4	56,633	206	275	13,804	223,958	72,740	357	0	1,342	311	18,470	13,964	961
Method 5	17,773	389	46	878	67,469	2,756	6,346	878	88	0	802	287	414
Method 6	14,776	101	146	7,715	304,075	14,824	105	0	192	78	3,696	2,730	296
Method 7	27,567	86	321	16,254	397,275	13,476	93	0	191	12	23,404	1,556	169
Method 8	11,157	26	429	1,244	407,159	10,680	1,320	0	38	4	1,359	2,756	77
Method 9	19,662	23	855	397	386,144	7,177	0	0	1,129	14	969	231	869
Method 10	30,513	47	629	843	533,095	17,178	59	0	666	136	3,876	4,616	332
