# Supplementary material for: Glycans affect DNA extraction and induce substantial differences in gut metagenomic studies
Source: Sci Rep. 2016 May 18;6:26276. doi: 10.1038/srep26276 (PMC4870698; doi:10.1038/srep26276)
Supplement: Supplementary Information [file srep26276-s1.pdf]

Running head: Illumina MiSeq deep sequencing and DNA extraction

Glycans affect DNA extraction and induce dramatic differences in gut metagenomic studies

Emmanouil Angelakis[1], Dipankar Bachar[1], Bernard Henrissat[2,3], Fabrice Armougom[1], Gilles Audoly[1], Jean-Christophe Lagier[1], Catherine Robert[1] and Didier Raoult[1*]

[1]URMITE CNRS-IRD 198 UMR 6236, Université de la Méditerranée, Faculté de Médecine, 27 Bd Jean Moulin, 13385 Marseille, France

[2]Centre National de la Recherche Scientifique, UMR 7257, Aix-Marseille Université, Marseille, France

[3]Department of Biological Sciences, Faculty of Science, King Abdulaziz University, Jeddah, Saudi Arabia

[*] Corresponding author

Supplementary table 1. The number of OTUs for all reads for each extraction method

DNA extraction method	No of high quality reads	No. of OTUs (cutoff distance unit 3)	Chao (cutoff distance unit 3)	Non parametric shannon (cutoff distance unit 3)
**Method 1**	49,719	683	927	2.46
**Method 1b**	13,016	320	448	2.42
**Method 2**	21,828	245	321	2.41
**Method 2b**	6,449	163	227	2.73
**Method 3**	35,648	437	609	1.37
**Method 3b**	28,524	291	342	1.62
**Method 4**	117,228	1,555	2,396	3.03
**Method 4b**	81,242	1,170	1,615	3.09
**Method 5**	46,059	1,841	2,714	3.81
**Method 5b**	26,629	1,762	2,629	4.40
**Method 6**	74,516	805	1,055	2.44
**Method 6b**	99,109	585	935	1.64
**Method 7**	9,322	231	344	2.31
**Method 7b**	27,693	463	606	2.60
**Method 8**	47,104	202	280	1.49
**Method 8b**	33,602	198	279	1.56
**Method 9**	273,155	2,127	2,349	3.79
**Method 9b**	192,833	798	1,239	2.93
**Method 10**	259,861	998	1,479	3.17

Supplementary Table 2. Pearson correlation coefficient

	Method1	Method2	Method3	Method4	Method5	Method6	Method7	Method8	Method9
**Method2**	0.497								
**Method3**	0.179	0.046							
**Method4**	0.705	0.510	0.131						
**Method5**	0.426	0.186	0.079	0.293					
**Method6**	0.994	0.519	0.181	0.681	0.425				
**Method7**	0.927	0.766	0.151	0.665	0.386	0.933			
**Method8**	0.959	0.663	0.164	0.640	0.407	0.978	0.969		
**Method9**	0.695	0.950	0.097	0.655	0.275	0.714	0.876	0.820	
**Method10**	0.975	0.618	0.169	0.738	0.415	0.987	0.952	0.986	0.80

Supplementary Table 3. Genera and species detection by the different extraction methods for the obese stool sample

DNA extraction method	Genera detected	%	Species detected	%
**Method 1**	68	20	111	15
**Method 2**	62	18	100	13
**Method 3**	110	33	155	19
**Method 4**	98	29	181	24
**Method 5**	258	77	522	70
**Method 6**	107	32	175	23
**Method 7**	55	16	98	13
**Method 8**	78	23	109	15
**Method 9**	47	14	103	14
**Method 10**	70	21	159	21
**Total**	336		746	

Supplementary Table 4. Results obtained for the 83 stool samples

	Extraction Method 1							Extraction Method 5										
**Sample**	**No of reads**	**No of genera**	**No of unique genera**	**No of species**	**No of unique species**	**chao**	**shannon**	**No of reads**	**No of genera**	**No of unique genera**	**No of species**	**No of unique species**	**chao**	**shannon**	**No of common genera**	**Total different genera**	**No of common species**	**Total species**
Amz 1	90,626	94 (83%)	20	327 (77%)	172	9,886	10.1	68,798	95 (84%)	21	252 (59%)	97	5,914	9.8	72	113	155	424
Amz 2	155,241	84 (88%)	16	312 (74%)	135	10,386	10.4	82,262	82 (85%)	15	287 (68%)	110	6,873	9.9	65	96	177	422
Amz 3	118,115	71 (85%)	13	232 (69%)	94	7,640	10.2	100,749	74 (88%)	16	243 (72%)	105	7,518	10.0	55	84	138	337
Amz 4	150,169	79 (100%)	25	240 (90%)	145	9,509	10.3	34,834	56 (71%)	3	123 (46%)	28	2,363	9.0	51	79	95	268
Amz 5	108,277	84 (85%)	11	276 (75%)	110	9,430	10.2	96,562	90 (91%)	17	258 (70%)	92	8,152	10.1	71	99	166	368
Amz 6	109,734	83 (90%)	24	218 (77%)	123	8,873	10.2	48,885	70 (76%)	11	160 (57%)	65	4,126	9.5	57	92	95	283
Amz 7	76,466	86 (77%)	20	263 (67%)	128	6,850	9.8	67,927	94 (87%)	27	265 (67%)	130	5,030	9.6	65	112	135	393
Amz 8	88,878	103 (87%)	29	280 (71%)	154	7,392	10.0	87,456	92 (78%)	18	241 (61%)	115	6,257	9.9	71	118	126	395
Amz 9	90,592	84 (70%)	18	221 (63%)	110	8,078	10.1	57,914	105 (88%)	39	243 (69%)	132	5,289	9.6	63	120	111	353
Amz 10	67,421	89 (83%)	21	278 (74%)	125	7,750	9.9	50,012	89 (83%)	21	250 (67%)	97	6,061	9.6	65	107	153	375
Amz 11	53,035	78 (76%)	12	214 (64%)	75	5,488	9.6	60,693	93 (90%)	27	262 (78%)	123	6,073	9.7	64	103	139	337
Amz 12	60,772	86 (91%)	16	281 (73%)	129	6,727	9.8	70,205	80 (84%)	11	256 (66%)	104	7,322	9.9	68	95	152	385
Amz 13	77,660	67 (74%)	9	173 (62%)	51	6,719	9.9	100,540	85 (93%)	26	227 (82%)	105	8,409	10.1	56	91	122	278
Amz 14	104,404	82 (81%)	11	242 (67%)	79	7,327	10.0	115,435	93 (92%)	21	284 (78%)	121	9,131	10.2	69	101	163	363
Amz 15	72,511	95 (76%)	13	398 (68%)	117	7,767	9.9	92,249	115 (92%)	32	470 (80%)	189	10,248	10.2	80	125	281	587
Amz 16	98,980	101 (72%)	22	288 (57%)	103	6,826	9.9	116,581	121 (86%)	42	400 (80%)	215	9,135	10.2	76	140	185	503
Amz 17	72,908	85 (80%)	14	268 (59%)	112	7,351	9.9	87,640	95 (90%)	22	341 (75%)	185	7,935	10.0	70	106	156	453
Amz 18	98,807	156 (83%)	42	538 (75%)	249	12,157	10.3	94,040	149 (79%)	35	499 (67%)	210	11,427	10.3	111	188	289	748
Amz 19	106,771	144 (78%)	42	390(65%)	178	10,788	10.3	128,965	145(79%)	42	419 (70%)	207	10,758	10.3	100	184	212	597
Amz 20	149,239	133 (90%)	32	598 (80%)	266	16,030	10.6	81,974	119 (80%)	17	480 (64%)	148	9,355	10.1	99	148	332	746
Amz 21	90,174	69 (68%)	18	224 (66%)	137	7,504	10.0	145,812	87 (85%)	34	200 (59%)	113	5,131	10.4	50	102	87	337
Amz 22	122,802	103 (84%)	31	356 (77%)	193	12,773	10.4	93,144	94 (76%)	22	271 (58%)	108	8,151	10.1	70	123	163	464
Amz 23	63,779	94 (74%)	35	224 (52%)	133	5,844	9.7	95,622	95 (75%)	35	299 (69%)	208	8,024	10.1	57	127	91	432
Toua 1	113,096	102 (83%)	19	389 (72%)	168	11,959	10.4	126,936	106 (86%)	22	371 (69%)	150	11,331	10.4	82	123	221	539

Toua 2	189,245	49 (60%)	6	227 (60%)	62	5,309	10.6	136,335	78 (95%)	34	318 (84%)	153	6,857	10.1	42	82	165	380
Toua 3	128,265	134 (84%)	34	604 (77%)	266	12,639	10.5	72,344	127 (80%)	27	516 (66%)	178	7,270	9.9	98	159	338	782
Toua 4	59,244	84 (77%)	10	298 (59%)	78	4,596	9.5	101,526	100 (92%)	26	429 (85%)	209	8,290	10.1	73	109	220	507
Toua 5	22,961	117 (84%)	63	408 (67%)	303	1,376	8.7	51,442	79 (56%)	26	305 (50%)	200	2,318	9.2	51	140	105	608
Toua 6	68,431	56 (82%)	11	211 (69%)	73	1,913	9.3	86,262	58 (85%)	13	233 (76%)	95	2,308	9.5	44	68	138	306
Fr 1	105,258	65 (83%)	14	238 (74%)	92	4,551	9.8	101,501	66 (85%)	14	229 (71%)	83	4,202	9.8	50	78	146	321
Fr 2	95,133	69 (76%)	16	330 (73%)	153	9,154	10.1	120,015	77 (85%)	23	297 (67%)	120	8,251	10.2	52	91	177	450
Fr 3	161,345	60 (80%)	10	230 (77%)	97	8,609	10.3	87,425	67 (89%)	17	200 (74%)	67	4,743	9.8	48	75	133	297
Fr 4	77,332	71 (83%)	15	276 (72%)	124	7,202	9.9	84,123	73 (85%)	16	262 (68%)	110	6,030	9.8	55	86	152	386
Fr 5	128,584	84 (87%)	14	451 (80%)	149	11,189	10.4	133,213	85 (88%)	14	415 (73%)	113	9,894	10.3	69	97	302	564
Fr 6	74,170	64 (88%)	11	311 (71%)	93	7,547	10.0	119,462	63 (86%)	10	348 (79%)	130	8,723	10.2	52	73	218	441
Fr 7	81,787	97 (87%)	29	387 (75%)	188	12,051	10.2	97,057	85 (76%)	16	326 (63%)	127	10,307	10.1	67	112	199	514
Fr 8	59,206	102 (82%)	36	421 (57%)	231	5,758	9.7	140,386	90 (72%)	25	504 (48%)	314	10,642	10.4	64	125	190	735
Fr 9	103,435	89 (74%)	41	371 (64%)	303	3,643	9.6	82,532	80 (67%)	32	279 (80%)	211	6,816	9.8	47	120	68	582
Fr 10	99,167	81 (68%)	27	340 (55%)	208	9,938	10.2	96,112	93 (78%)	39	406 (66%)	274	5,800	9.9	53	119	132	614
Fr 11	88,733	87 (71%)	38	303 (48%)	221	3,510	9.5	153,633	85 (70%)	36	415 (65%)	333	12,881	10.5	48	122	82	636
Fr 12	77,213	85 (69%)	34	401 (61%)	303	7,080	10.0	96,364	91 (73%)	40	355 (54%)	257	4,083	9.6	50	124	98	658
Fr 13	112,622	60 (83%)	10	250 (73%)	100	2,616	9.5	87,630	63 (88%)	13	241 (71%)	91	2,259	9.3	49	72	150	341
Fr 14	80,740	54 (70%)	9	186 (65%)	66	4,554	9.8	79,493	69 (90%)	24	221 (77%)	101	4,479	9.7	44	77	120	287
Fr 15	163,474	72 (89%)	20	402 (87%)	230	9,068	10.4	83,726	62 (77%)	10	231 (50%)	59	5,298	9.9	51	81	172	461
Fr 16	82,488	88 (78%)	12	393 (74%)	122	8,801	10.1	102,575	103 (91%)	27	412 (77%)	141	9,432	10.2	74	113	271	534
Fr 17	78,410	94 (75%)	10	461 (69%)	156	7,306	9.9	114,776	116 (93%)	32	508 (77%)	203	9,225	10.2	83	125	305	664
Fr 18	105,010	56 (86%)	16	202 (77%)	104	4,347	9.8	106,352	51 (78%)	10	159 (60%)	61	3,388	9.7	39	65	98	263
Fr 19	105,040	79 (81%)	13	372 (76%)	154	8,171	10.1	98,453	86 (88%)	20	338 (69%)	120	5,710	9.9	65	98	218	492
Fr 20	85,774	99 (84%)	25	479 (70%)	160	10,851	10.3	123,600	94 (80%)	20	521 (77%)	202	13,333	10.5	73	118	319	681
Fr 21	108,470	83 (90%)	19	313 (80%)	133	9,363	10.3	73,133	74 (80%)	10	256 (66%)	76	6,183	9.9	63	92	180	389
Fr 22	121,942	60 (79%)	13	268 (67%)	117	6,185	10.1	117,868	65 (86%)	18	283 (71%)	132	6,551	10.1	45	76	151	400
Fr 23	94,202	95 (89%)	19	337 (74%)	122	5,574	9.8	109,750	89 (83%)	13	332 (73%)	117	6,525	9.9	75	107	215	454
Fr 24	89,204	105 (78%)	23	478 (70%)	141	9,425	10.1	131,223	112 (84%)	30	538 (79%)	201	12,184	10.5	81	134	337	679
Fr 25	109,415	95 (84%)	17	510 (79%)	204	11,708	10.3	101,583	97 (86%)	19	442 (68%)	136	9,207	10.2	77	113	306	646
Fr 26	114,955	71 (86%)	25	324 (83%)	160	10,376	10.4	50,781	59 (71%)	13	232 (59%)	68	4,461	9.6	45	83	164	392

Fr 27	69,356	78 (80%)	22	273 (76%)	167	3,230	9.4	52,467	76 (78%)	20	191 (53%)	85	2,384	9.3	55	97	106	358
Fr 28	64,505	69 (78%)	10	299 (78%)	120	5,444	9.8	59,235	80 (90%)	21	264 (69%)	85	4,709	9.7	58	89	179	384
Fr 29	98,831	60 (88%)	22	198 (86%)	125	3,144	9.6	40,666	47 (69%)	10	106 (56%)	33	1,034	8.7	36	68	73	231
Fr 30	122,330	82 (85%)	18	298 (68%)	116	10,981	10.4	127,426	80 (82%)	16	320 (73%)	138	10,361	10.4	63	97	182	436
Fr 31	113,181	74 (88%)	10	366 (80%)	133	10,899	10.3	58,533	75 (89%)	11	327 (71%)	94	7,402	9.8	63	84	233	460
Fr 32	80,636	66 (73%)	14	284 (63%)	130	3,175	9.3	70,356	77 (86%)	25	324 (71%)	170	5,952	9.9	51	90	154	454
Fr 33	94,873	82 (92%)	26	343 (86%)	204	4,996	9.6	48,678	64 (72%)	8	193 (49%)	54	2,715	9.1	55	89	139	397
Mrm 1	44,683	31 (53%)	2	108 (47%)	37	1,084	8.9	199,143	58 (98%)	29	191 (84%)	120	1,911	10.0	28	59	71	228
Mrm 2	36,948	43 (84%)	17	168 (66%)	85	1,510	8.9	191,811	35 (69%)	9	170 (67%)	87	1,031	9.9	25	51	83	255
Mrm 3	26,193	35 (51%)	6	143 (55%)	58	857	8.5	186,642	64 (93%)	35	200 (78%)	115	1,499	10.1	28	69	85	258
Mrm 4	26,734	35 (71%)	9	98 (52%)	32	859	8.4	243,486	41 (84%)	15	156 (83%)	90	2,177	10.3	25	49	66	188
Mrm 5	30,610	33 (66%)	4	81 (41%)	35	1,102	8.6	225,582	47 (94%)	18	164 (82%)	118	1,688	10.2	28	50	46	199
Mrm 6	45,648	36 (86%)	6	112 (64%)	50	1,385	9.0	197,824	37 (88%)	7	125 (71%)	63	1,749	10.3	29	42	62	175
Mrm 7	37,147	38 (63%)	7	93 (50%)	24	1,593	9.0	167,845	54 (90%)	23	162 (87%)	93	3,240	9.9	30	60	69	186
Mrm 8	53,225	46 (81%)	19	151 (72%)	74	1,015	8.8	154,437	39 (68%)	12	137 (65%)	60	923	9.5	26	57	77	211
Mrm 9	49,525	57 (83%)	12	220 (75%)	87	2,502	9.2	131,416	58 (84%)	13	206 (70%)	73	2,622	9.6	44	69	133	293
KW 1	61,269	59 (77%)	16	173 (70%)	89	1,623	9.0	189,864	62 (81%)	19	159 (64%)	75	1,291	10.1	42	77	84	248
KW 2	55,189	48 (77%)	15	168 (73%)	75	925	8.9	189,727	48 (77%)	15	154 (67%)	61	1,034	10.1	32	62	93	229
KW 3	45,271	24 (43%)	5	98 (54%)	36	503	8.9	211,872	52 (93%)	32	147 (80%)	85	1,040	10.2	19	56	62	183
KW 4	80,949	64 (75%)	22	232 (66%)	115	2,155	9.5	154,792	64 (75%)	22	238 (67%)	121	2,584	9.9	41	85	117	353
KW 5	35,945	44 (76%)	10	107 (66%)	49	977	8.7	98,025	49 (84%)	16	114 (70%)	56	1,442	9.4	32	58	58	163
KW 6	34,430	42 (89%)	11	137 (74%)	76	1,191	8.7	52,922	37 (79%)	6	109 (59%)	48	695	8.8	30	47	61	185
KW 7	25,6815	37 (82%)	12	188 (85%)	96	2,571	10.3	51,339	34 (76%)	9	124 (56%)	32	1,207	8.8	24	45	92	220
KW 8	19,4823	38 (97%)	16	190 (88%)	121	1,974	10.2	33,137	24 (62%)	2	95 (44%)	26	646	8.6	21	39	69	216
KW 9	16,4638	85 (98%)	45	464 (95%)	368	5,093	10.1	26,462	43 (49%)	3	120 (25%)	24	797	8.4	39	87	96	488
KW 10	23,3191	65 (98%)	30	304 (92%)	194	2,371	10.3	33,530	37 (56%)	2	135 (41%)	25	718	8.7	34	66	110	329
Ob1	49,310	59 (69%)	10	264 (60%)	59	3,647	7.9	66,626	75 (88%)	26	376 (86%)	171	5,980	10.3	49	85	208	438
Ob2	64,775	63 (90%)	14	262 (76%)	104	3,598	8.0	52,324	56 (84%)	7	244 (71%)	83	2,930	9.8	49	70	158	345

Amz, individuals from Amazone; Toua, Touareg individuals; Fr, French individuals; Mrm, Marasmus individuals; KW, kwashiorkor individuals; Ob, obese individuals

Supplementary Table 5. Percentage of detection by the two extraction assays for the 30 most common genera

Genera	Extraction method 1	Extraction method 5
Acidaminococcus	37	63
Acinetobacter	78	22
Actinomyces	26	74
Aeromonas	13	87
Anaerostipes	61	39
Atopobium	23	77
Bifidobacterium	31	69
Collinsella	25	75
Enterobacter	64	36
Enterococcus	38	62
Enterorhabdus	27	73
Faecalibacterium	65	35
Hafnia	6	94
Klebsiella	61	39
Leuconostoc	19	81
Megamonas	38	62
Megasphaera	37	63
Mycobacterium	16	84
Oribacterium	33	67
Paraprevotella	39	61
Phascolarctobacterium	38	62
Prevotella	58	42
Psychrobacter	69	31
Sarcina	33	67
Slackia	37	63
Sporosarcina	0	100
Streptococcus	42	58
Sutterella	62	38
Treponema	67	33
Weissella	32	68

Supplementary Figure 1. Number of sequence reads obtained by each DNA extraction with and without normalization.

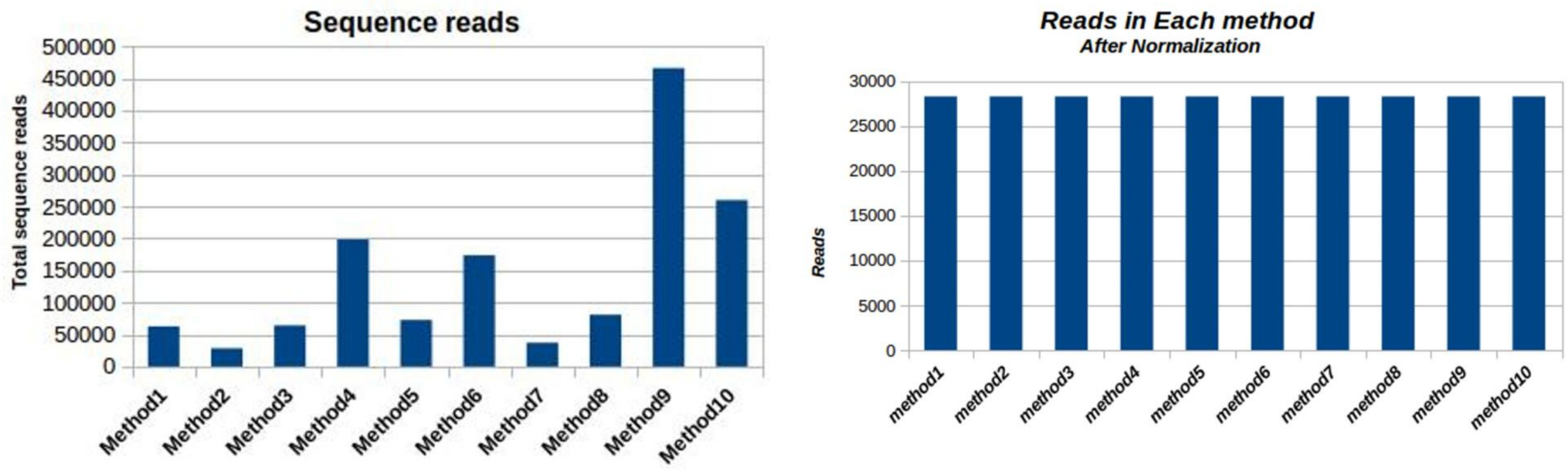

Supplementary figure 2. Rarefaction curves showing the number of OTUs produced by each method

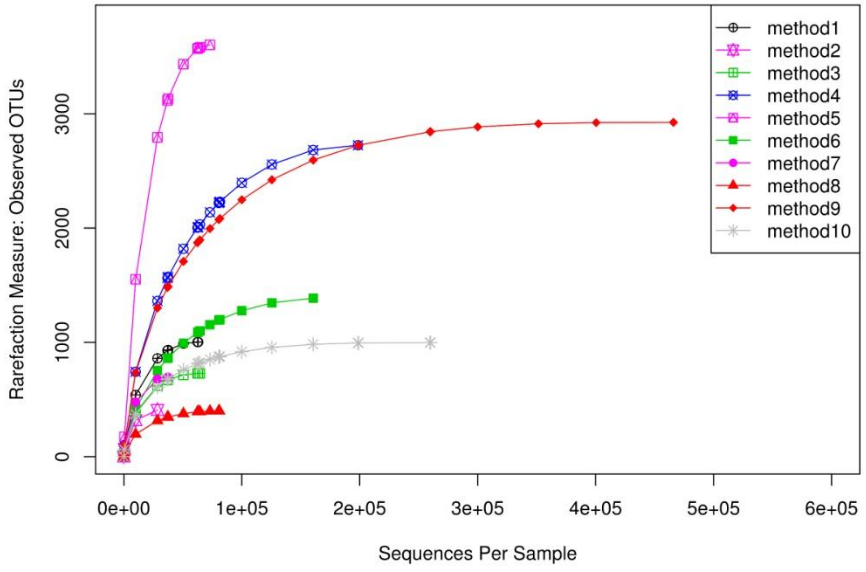

Without Normalization

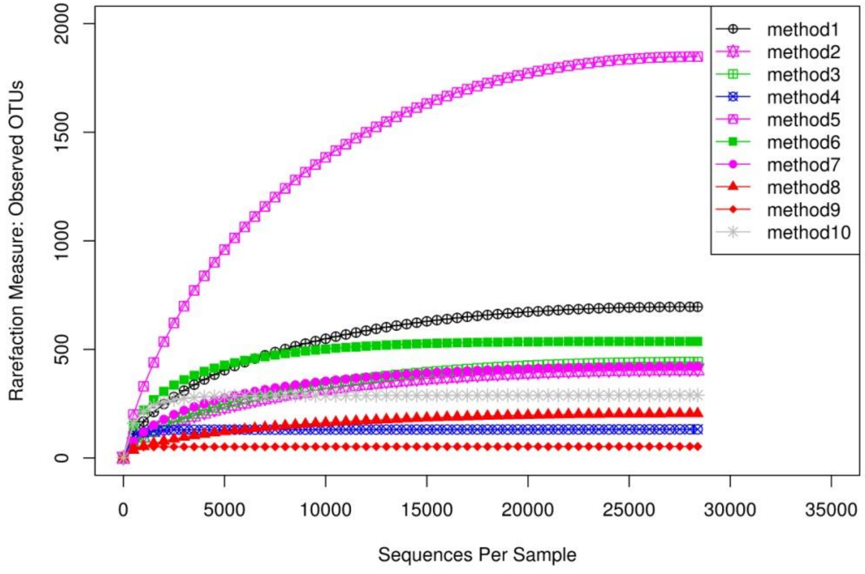

With Normalization

Supplementary figure 3. Principal coordinate analysis comparison of microbial community compositions following DNA extraction using different methods; A, before and B, after normalization.

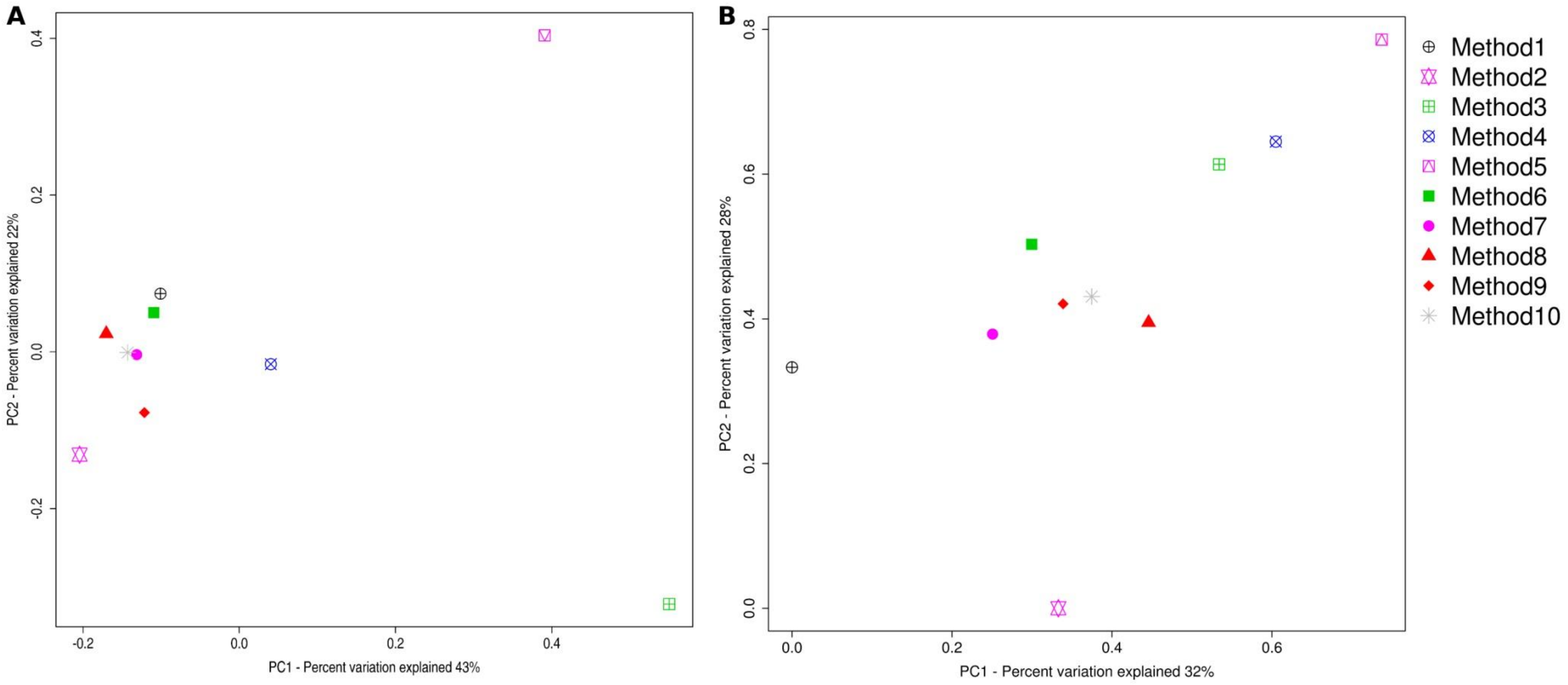

Supplementary figure 4. Principal component analysis comparison of microbial community compositions following DNA extraction using different methods; A, before and B, after normalization.

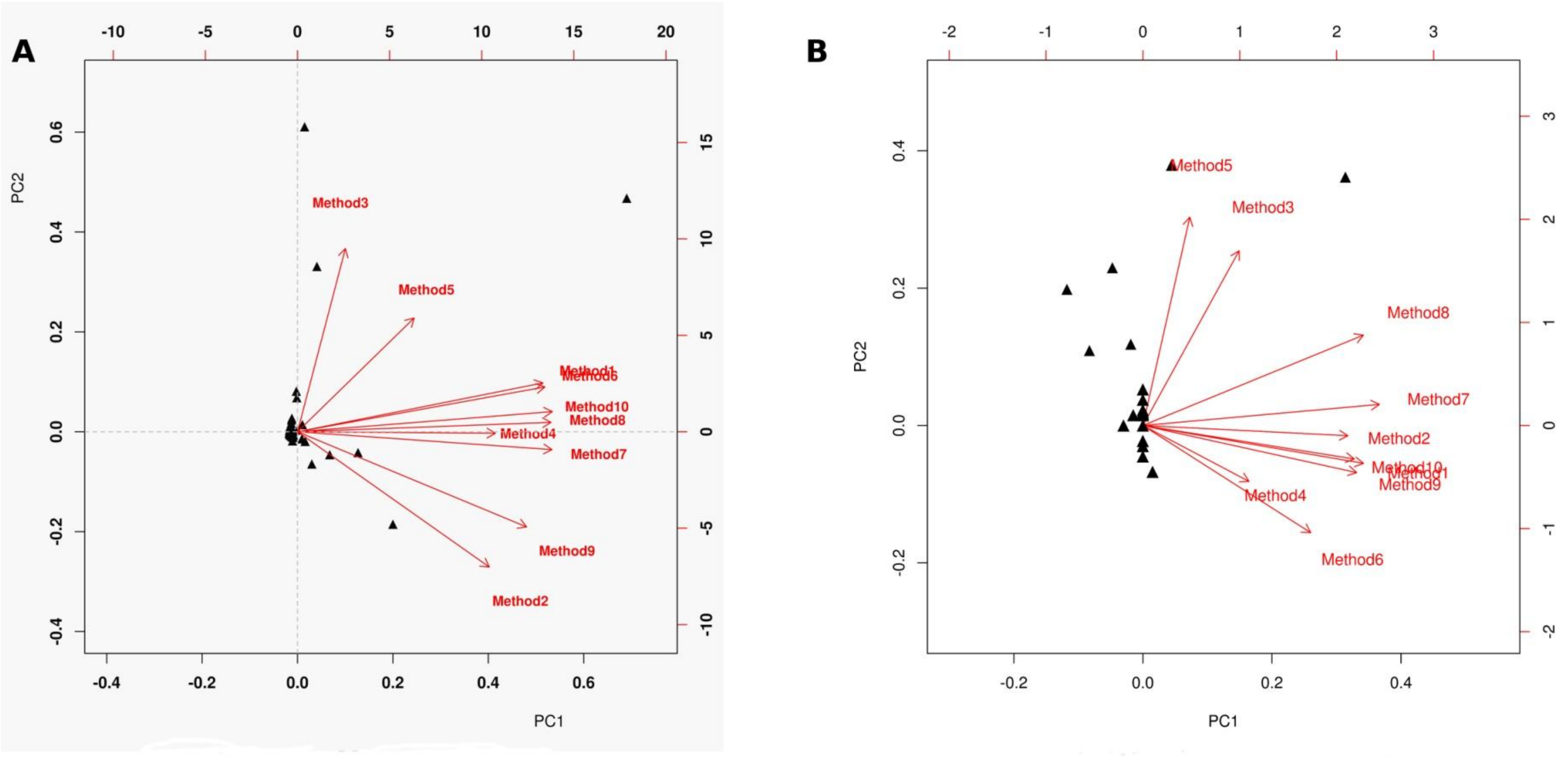

Supplementary figure 5. Phylum relative abundance found by different extraction methods.

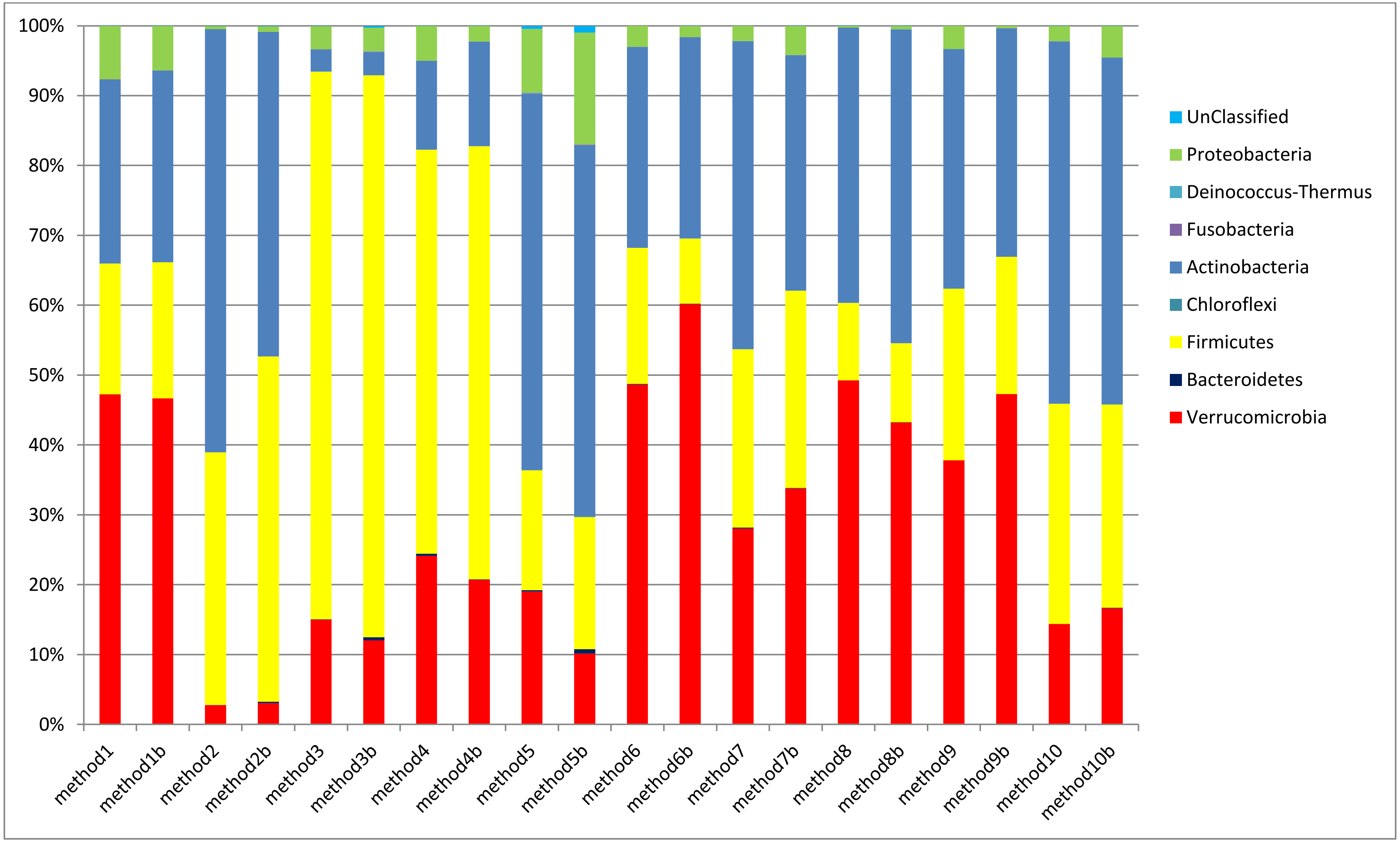

Supplementary figure 6. Number of phyla detected by the extraction method 1 and 5 for the 83 stool samples

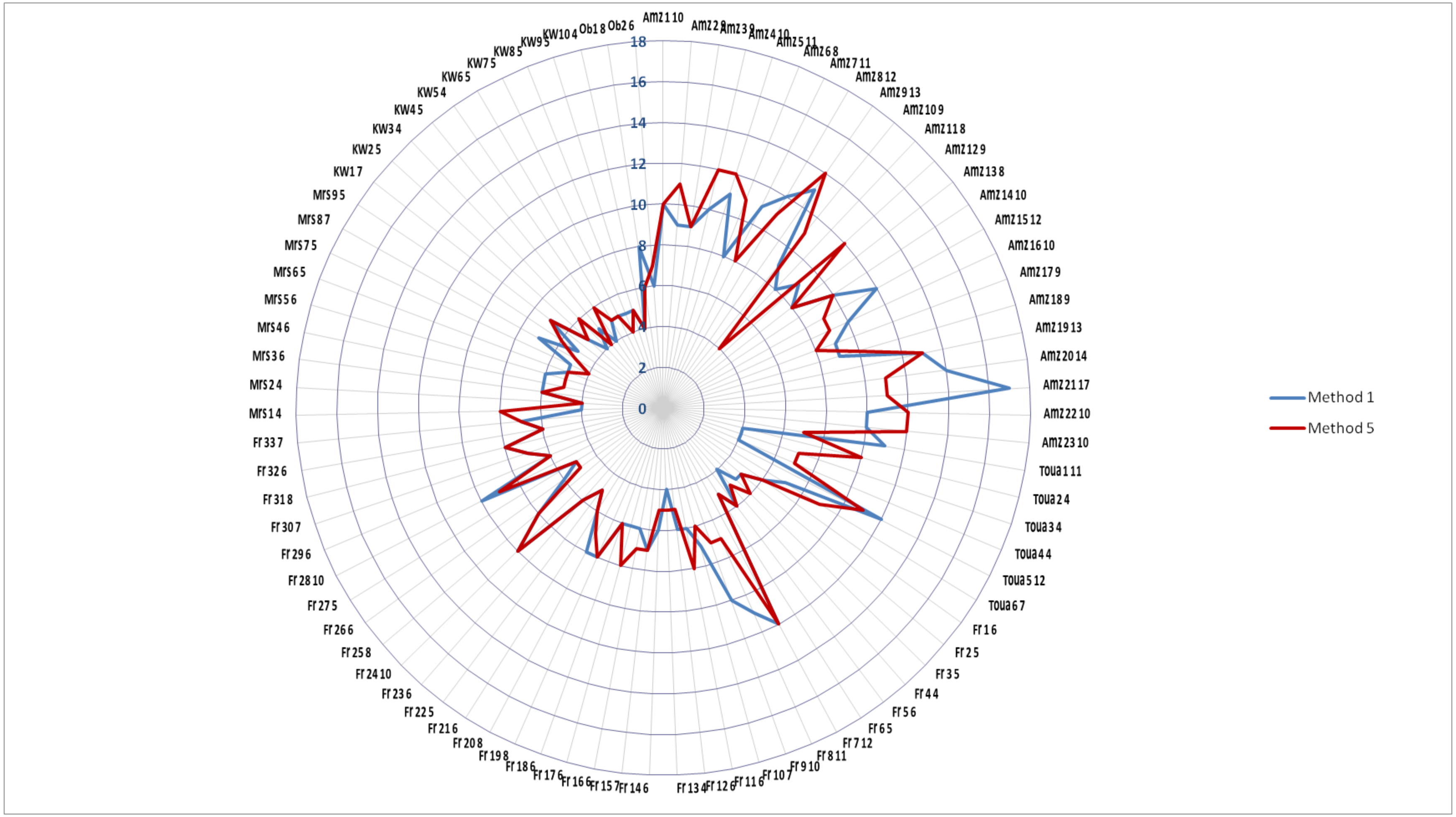

Amz, individuals from Amazone; Toua, Touareg individuals; Fr, French individuals; Mrm, Marasmus individuals; KW, kwashiorkor individuals; Ob, obese individuals

Supplementary figure 7. Number of genera detected by the extraction method 1 and 5 for the 83 stool samples

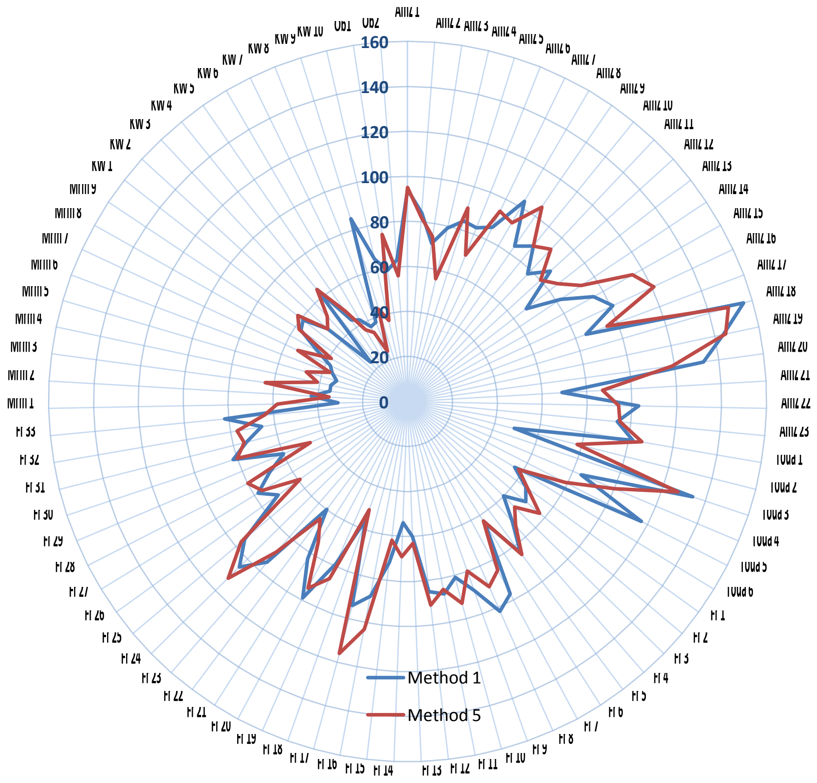

Amz, individuals from Amazone; Toua, Touareg individuals; Fr, French individuals; Mrm, Marasmus individuals; KW, kwashiorkor individuals; Ob, obese individuals

Supplementary figure 8. Phylum relative abundance found by the extraction method 1 and 5 for the three obese individuals.

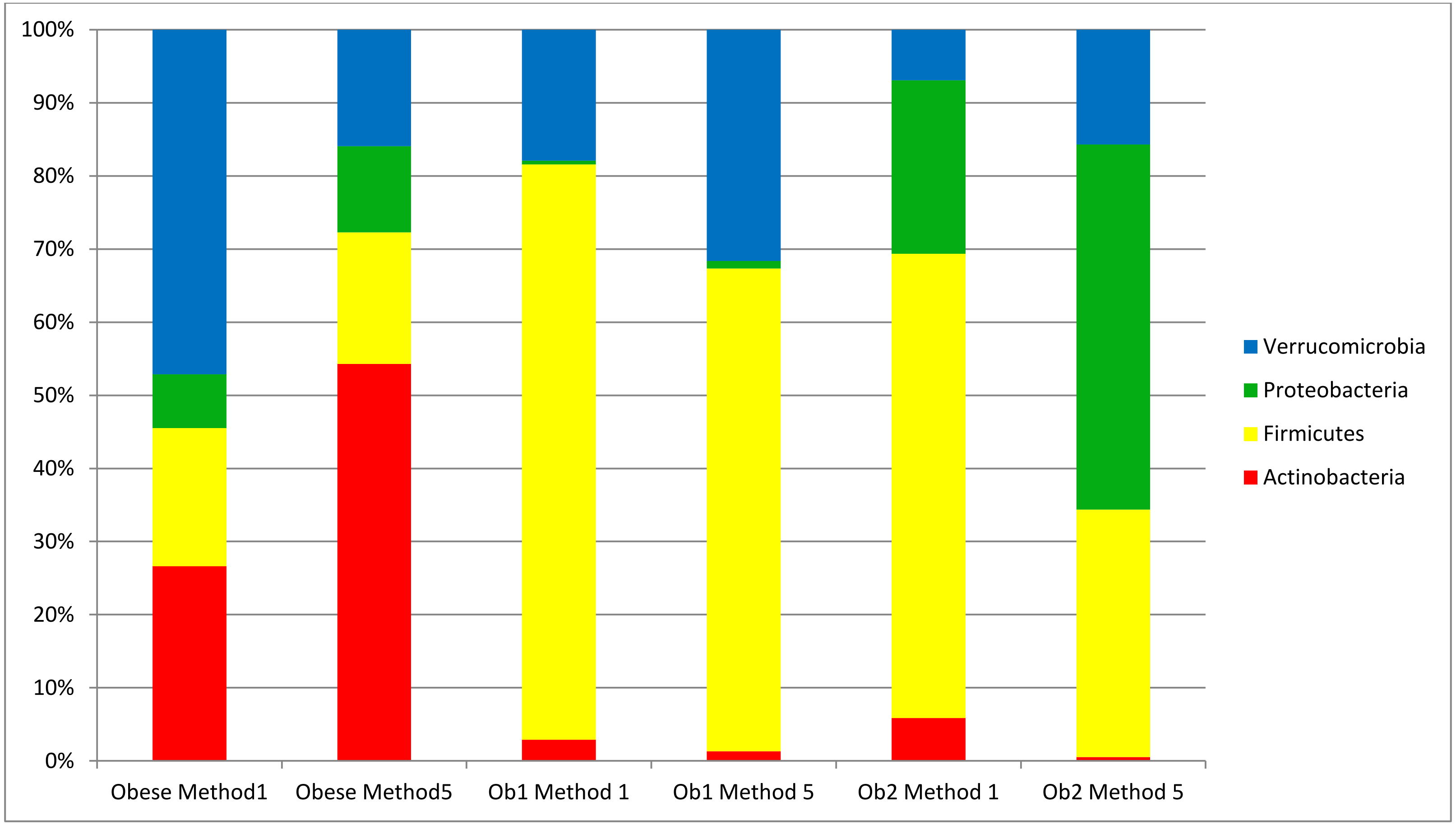

Ob, obese individual

Supplementary figure 9. Number of species detected by the extraction method 1 and 5 for the 83 stool samples

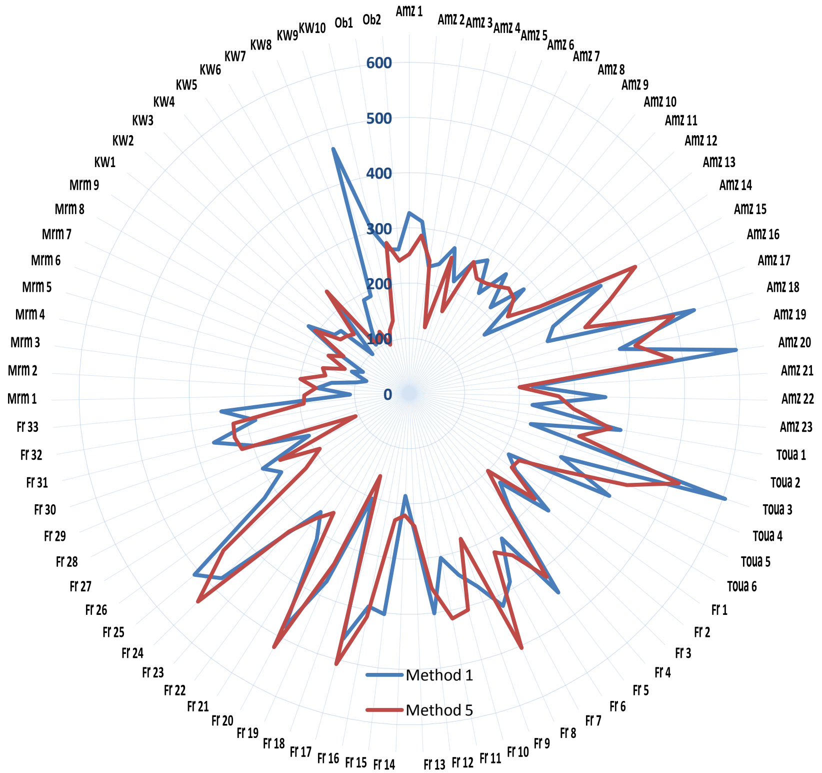

Amz, individuals from Amazone; Toua, Touareg individuals; Fr, French individuals; Mrm, Marasmus individuals; KW, kwashiorkor individuals; Ob, obese individuals
